# The relationship between physical activity, self-efficacy and quality of life in colorectal cancer survivors: a multicenter cross-sectional study

**DOI:** 10.1007/s12672-023-00854-5

**Published:** 2024-01-05

**Authors:** Yuru Hu, Lijun Wang, Guoqiang Su, Bo Chen, Zheng Ruan, Jinqiu Yang, Qu Shen

**Affiliations:** 1 School of Medicine, Xiamen University, Xiangshan Street, Xiang’an District, Xiamen, 361102 Fujian China; 2 Department of Colorectal Surgery, The First Affiliated Hospital of Xiamen University, 55 Zhenhai Road, Xiamen, 361003 Fujian China; 3 Department of Nursing, School of Medicine, Xiamen University, Xiangshan Street, Xiang’an District, Xiamen, 361102 Fujian China

**Keywords:** Cancer, Oncology, Colorectal cancer survivors, Physical activity, Self-efficacy, Quality of life, Cross-sectional study

## Abstract

**Purpose:**

This study aimed to investigate the current situation and factors influencing physical activity, self-efficacy, and quality of life in Chinese colorectal cancer survivors. Additionally, this study explored the associations between physical activity, self-efficacy, and quality of life.

**Methods:**

A multicenter, cross-sectional study was conducted, involving 173 colorectal cancer survivors with a mean age of 59 years. Self-reported data on basic demographic characteristics, physical activity, self-efficacy, and quality of life were collected.

**Results:**

Among 173 colorectal cancer survivors, 90 (52.0%) were engaged in manual work. The self-efficacy score was found to be 25.99 ± 7.10, while the global health status score was 54.96 ± 21.56. Global health status was associated with sex, residence, chemoradiotherapy, and monthly income (p < 0.01). The self-efficacy score exhibited a significant positive correlation with quality of life, while demonstrating a negative correlation with symptom scores (p < 0.01). Recreational PA scores were positively associated with global health status (P < 0.05). Self-efficacy, recreational physical activity during winter, and whether the participants underwent chemoradiotherapy explained 29.3% of the variance in quality of life among colorectal cancer survivors.

**Conclusions:**

Colorectal cancer survivors exhibited low levels of physical activity, self-efficacy, and quality of life. Their health is influenced by self-efficacy, recreational physical activity, and chemoradiotherapy. When developing intervention plans for colorectal cancer survivorship, it is crucial to consider survivors' self-efficacy and the type of physical activity in which they engage.

## Introduction

According to epidemiological surveys, the global number of new cancer cases reached approximately 19,292,789, with 9,958,133 cancer-related deaths reported in 2020 [1]. Colorectal cancer (CRC) was estimated to account for 1.93 million new cancer cases worldwide in 2020, ranking third in terms of morbidity rate and second in terms of mortality rate globally, according to the World Health Organization (WHO) [2]. CRC is also the 3rd most morbidity among males and the 2nd most morbidity among females [2], posing a significant global public health concern. As advancements in medical technology continue, the survival rate of cancer patients has substantially increased [3], leading to a rise in the number of CRC survivors.

CRC survivors often suffer from persistent symptoms and dysfunctions following treatment, such as diarrhea, constipation, fatigue, and sexual dysfunction, which can be attributed to surgical procedures and side effects of chemoradiotherapy [4, 5]. Additionally, they may experience severe psychosocial impacts [6]. Therefore, CRC survivors tend to have a lower quality of life (QoL) [7, 8]. Consequently, promoting their health-related quality of life (HRQoL) has become increasingly important.

Evidence suggests that moderate physical activity (PA) can have positive effects on health outcomes and quality of life (QoL) for CRC survivors [9]. The WHO defines physical activity (PA) as any bodily movement produced by skeletal muscles that requires energy expenditure [10]. As a non-pharmacological modality, PA has been shown to improve survival outcomes [11]. However, self-reported levels of PA among CRC survivors often fall short of the recommended 150 min of moderate-to-vigorous PA per week [12]. In a study conducted in China, which examined the PA status of 174 CRC survivors undergoing chemotherapy, it was found that only 7.5% of survivors engaged in sufficient PA, while 32.2% were completely sedentary [13]. Another study by Krogsgaard reported that only half of the patients with long-term stoma adhered to the WHO guideline recommendation for PA [11]. Therefore, it is important to explore the factors influencing PA among CRC survivors in order to enhance their PA levels.

Self-efficacy has been proven to be a key determinant related to the adoption and maintenance of PA among individuals with chronic diseases such as cancer, stroke, diabetes, and heart disease. Notably, there exists a significant positive correlation between self-efficacy scores and PA levels [12]. Self-efficacy is also considered a psychosocial determinant of PA in cancer patients, and improving self-efficacy can significantly improve compliance [14]. Furthermore, higher levels of self-efficacy have been consistently linked to enhanced quality of life (QoL) outcomes [15].

The aim of our study was to investigate the factors influencing QoL, self-efficacy, and PA levels in CRC survivors, as well as exploring the interrelationships among these factors. The findings of this study aim to establish a theoretical foundation for developing effective PA plans and guidelines that can enhance the QoL of CRC survivors.

## Methods

### Study design and setting

The study was designed as a multicenter cross-sectional survey conducted between January and May 2021. CRC survivors were recruited from four departments (medical oncology, surgical oncology, interventional oncology, and radiotherapy) in three tertiary hospitals in Xiamen City (the highest-level hospital in China). Convenience sampling was utilized to select participants. The sample size was calculated according the formula: N = [Uασ/δ]2, with Uα = 1.96, δ = 0.1. Referring to the findings by HUANG Yu [16], σ = 0.61. Based on the sample size calculation, the total number of survivors was 143. After allowing 20% attrition, the sample size increased to 171. Informed consent was obtained before participation in the study. Ethical approval was obtained from the Xiamen University School of Medical Research Ethics Board (Approval No. XDYX2021030, November 8, 2021).

### Participants

The participants in this study were CRC survivors who had received treatment at three hospitals in China. The eligibility criteria were as follows: (a) age ≥ 18 years, (b) postoperative CRC survivors, and (c) able to complete the survey. Survivors’ exclusion criteria included the presence of severe physical, cognitive, and/or verbal impairments that would interfere with the patient’s ability to provide informed consent and survivors with an indwelling stoma. Written informed consent was obtained from all participants.

All survivors who fulfilled the inclusion criteria were contacted by a trained nurse and consecutively recruited from the participating institutions. All the study procedures were performed in accordance with the precepts of Good Clinical Practice and the Declaration of Helsinki.

### Procedures

To ensure consistency and minimize bias, all data collectors were trained before they began collecting data. After obtaining the Chief Nurse's approval, the research team screened eligible survivors according to the inclusion/exclusion criteria, explained the aim and methodology of the study to the survivors, and obtained their informed consent. For inpatients, the questionnaires were supervised by a trained nurse, and the questionnaires were collected immediately after completion. Non-hospitalized survivors were allowed to respond to the questionnaire via telephone if necessary. The study was conducted using a paper questionnaire, which took approximately 15 min to complete, consisting of four parts (basic demographic characteristics, PA, self-efficacy, and QoL). Before completing the questionnaires, the participants were given instructions on the use of the questionnaires. All questionnaires were completed by survivors. If participants were unable to do so, they were asked to assist with the completion of the questionnaires. Once completed, the questionnaires were manually checked for completeness and stored appropriately.

The following steps were taken to reduce bias. First, participants were left alone while completing the questionnaire to minimize any influence on their responses. Second, the data were checked immediately by a trained investigator after completion of the questionnaire to ensure data integrity. Finally, the data were entered by two people and carefully checked during the entry process to reduce human error. The data were then checked again for accuracy by a third reviewer.

### Measurements

*Basic demographic characteristics* Data collected included general information (e.g., sex, age, and ethnicity) and clinical data (time of diagnosis and postoperative treatment).

*PA* PA was assessed using a modified Chinese version of the European Prospective Investigation into Cancer and Nutrition PA Questionnaire (EPIC-PAQ). The EPIC-PAQ was developed by the European Nutrition and Cancer Prospective Cohort Research Organization [17]. The Chinese version of the EPIC-PAQ scale was translated from the original and included three dimensions and four questions on occupational PA, domestic PA, and leisure time PA [18]. There are nine items in the questionnaire. It divides the population into four categories based on work status and leisure time: sedentary, light PA, moderate PA, very heavy PA, and lots of PA. EPIC assigns metabolic equivalents (METs) to each PA.

*Self-Efficacy* Survivors’ self-efficacy was measured using the Chinese version of the General Self-Efficacy Scale (GSES), which is unidimensional. Internal consistency was 0.87 [19]. The GSES consists of ten items, measuring how the patient believes he or she is doing. Each item has four response options (1 = completely wrong, 2 = somewhat right, 3 = most right, 4 = completely right). The patient chose the most appropriate answer according to his or her actual conditions and feelings. The maximum score is 40, higher scores indicate higher self-efficacy [20].

*Quality of life* HRQoL was assessed using the Chinese version of the European Organization for Research and Treatment of Cancer Quality of Life Questionnaire Core 30 (EORTC QLQ-C30) [21]. The EORTC QLQ-C30 is widely used, and has good reliability and validity. The scale has been revised locally in China and has shown good reliability and validity [22]. It consists of five functional scales, nine symptom-specific subscales, and a global health status scale. The EORTC QLQ-C30 symptom scales range from 0 to 100, with higher scores indicating worse symptoms. The EORTC QLQ-C30 global health status scale ranges from 0 to 100, with higher scores indicating better HRQoL. The Functional Scale includes the subscales of physical, role, social, emotional, and cognitive functioning, ranging from 0 to 100, with higher scores indicating better HRQoL.

### Statistical analysis

The analysis of PA considered the type of occupation and the seasonal variation in PA levels. Self-efficacy scores were based on the summation of items from the GSES. EORTC QLQ-C30 scores were based on the summation of each dimension and were analyzed separately.

The data analysis was conducted using SPSS 25 with a significance level set at α = 0.05. The quantitative data included variables such as age, EPIC-PAQ, GSES scores, and EORTC QLQ-C30 scores. The qualitative data included general clinical data such as sex, residence.

The data analyses consisted of four components. The first component was descriptive analysis, where qualitative data were presented as percentage and quantitative data were expressed as mean (SD). The second component involved comparing similarities and differences using independent-sample t-test and one-way analysis of variance. This compared the difference in PA, QoL, and self-efficacy across different situations of CRC survivors. The third component involved correlational analyses. Pearson correlations were used to analyze the associations between HRQoL, self-efficacy, and PA. Lastly, regression analyses were conducted using multivariate linear regression models to analyze the data.

## Results

### Sample characteristics

In January 2021 and May 2021, 173 adults were invited to participate in the survey. All these surveys were complete and considered usable for this study before data analysis.

Survivors were predominantly older (≥ 41 years), with a mean age of 59 years (± 11 years). More men (59%) than women (41%) in the study. There was a preponderance of urban residents. Table 1 provides further details on the demographics of the participants.

**Table 1:** Demographic and medical characteristics of colorectal cancer survivors (January 2021–May 2021; N = 173)

Variable	M ± SD or n (%)
Sex	
Male	102(59.0)
Female	71 (41.0)
Age	
Total	59.25 ± 11.4
18 ~ 40	11 (6.4)
41 ~ 65	112(4.7)
≥ 66	50 (28.9)
Residence location	
Urban	109(63.0)
Rural	64 (37.0)
Marital status	
Married	170(98.3)
Not married	3 (1.7)
Education level, years	
< 6	55 (31.8)
6–9	55 (31.8)
9–12	27 (15.6)
> 12	36 (20.8)
Monthly household income	
< 1700	22 (12.7)
1701 ~ 5800	79 (45.7)
5801 ~ 15,000	60 (34.7)
> 15,000	12 ( 6.9)
Received chemoradiotherapy	
Yes	93 (46.2)
No	80 (53.8)
Time of illness, month	
1~3	73 (42.2)
4~6	51 (29.5)
≥ 7	49 (28.3)

### Physically active, self-efficacy and quality of life scores of the study participants

*Physically active* 90 survivors (52.0%) had manual work, 63 survivors (36.4%) had sedentary work, 16 survivors (9.2%) had standing occupation, and 4 survivors (2.4%) had heavy manual work. Non-occupational PA was 85.58 ± 85.69 MET-h/w in summer and was 81.32 ± 77.35 MET-h/w in winter. According to the level of PA, light to moderate activity was 19.95 ± 1.56 h/w in summer and 19.06 ± 1.38 h/w in winter. Moderate to vigorous activity was 4.01 ± 0.44 h/w in summer and 3.80 ± 0.41 h/w in winter. (More details are reported in Table 1). Only 69 (40%) survivors in summer and 67 (39%) in winter met the guideline recommended level of moderate intensity physical activity of 150 min/week [23].

*Self-efficacy* In this study, the total score of this scale was 25.99 ± 7.10 points, with a mean score of 2.60 ± 0.71 points.

*Quality of life* The global health status score was 54.96 ± 21.56. The functioning scale with the lowest mean score was the social functioning scale (70.13 ± 22.96), whereas the highest score was on the cognitive functioning scale (89.69 ± 13.85). All subscale mean scores were above 50. Table 2 shows the details in terms of specific categories.

**Table 2:** Physical activity, self-efficacy and quality of life scores of colorectal cancer survivors (January 2021–May 2021; N = 173)

Variable	M ± SD or n (%)
Physical activity	
Occupational activities	
Sedentary occupation^a^	63 (36.4)
Standing occupation^b^	16 (9.2)
Manual work^c^	90 (52.0)
Heavy manual work^d^	4 (2.4)
Physical activity score	
Summer housework physical activity	39.68 ± 55.71
Winter housework physical activity	38.52 ± 53.38
Summer recreational physical activity	45.89 ± 44.92
Winter recreational physical activity	42.80 ± 38.55
Non-occupational physical activity in summer	85.58 ± 85.69
Non-occupational physical activity in winter	81.32 ± 77.35
Light to moderate activity in summer	19.95 ± 1.56
Light to moderate activity in winter	19.06 ± 1.38
Moderate to vigorous activity in summer	4.01 ± 0.44
Moderate to vigorous activity in winter	3.80 ± 0.41
Self-efficacy	25.99 ± 7.10
Quality of life	
Somatic function	84.70 ± 17.77
Role function	74.76 ± 27.05
Emotional function	81.36 ± 18.86
Cognitive function	89.69 ± 13.85
Social function	70.13 ± 22.96
Tiredness	28.07 ± 20.00
Nausea and vomiting	8.29 ± 15.32
Pain	16.28 ± 17.79
Shortness of breath	7.32 ± 14.30
Insomnia	24.28 ± 25.19
Anorexia	16.76 ± 22.91
Constipation	10.79 ± 20.94
Diarrhea	11.56 ± 19.88
Economic hardship	29.29 ± 25.98
Global health status	54.96 ± 21.56

^a^Sedentary occupation: This type of work requires a majority of time being seated, such as working in an office

^b^Standing occupation: This type of work requires prolonged periods of standing or walking, but does not require significant physical exertion. Examples include salespersons, hairdressers, security guards, etc.

^c^Manual work: It primarily involves physically demanding tasks, including moving heavy objects and using tools. Occupations like plumbers, electricians, carpenters, etc., fall under this category

^d^Heavy manual work: It refers to extremely strenuous physical activities that involve lifting and carrying heavy objects. Examples include dock workers, miners, bricklayers, construction workers, etc.

### Comparison the scores of physical activity, self-efficacy and quality of life

In this study, significant differences were observed in the distribution of household PA scores based on sex, residence, education level, and monthly income (p < 0.05). Additionally, Recreational PA showed differences only in the distribution of monthly income (p < 0.05).

Self-efficacy was found to be associated with sex, residence, monthly income, and duration of the disease (p < 0.01). Sex was associated with global health status (p < 0.05), with men having higher global health status than women. Residence was also associated with global health status (p < 0.01), with urban survivors having higher global health status compared to rural survivors. Furthermore, Survivors who had a higher education background generally had a better global health status (p < 0.01). Non-chemoradiotherapy survivors had higher global health status scores than chemoradiotherapy survivors (p < 0.05). (Table 3 reports further details).

**Table 3:** Comparison the scores of physical activity, self-efficacy and quality of life (January 2021–May 2021; N = 173)

Variable	Summer housework physical activity	Winter housework physical activity	Summer recreational physical activity	Winter recreational physical activity	Self-efficacy	Global health status
Sex						
Male	31.43 ± 51.81	29.59 ± 47.14	48.96 ± 49.42	44.80 ± 40.63	27.09 ± 6.93	58.17 ± 20.71
Female	51.54 ± 59.26	51.34 ± 59.25	41.49 ± 37.42	39.93 ± 35.44	24.42 ± 7.08	50.35 ± 22.07
t	**− 2.366**	**− 2.577**	**1.076**	**0.816**	**2.466**	**2.377**
P	0.019*	0.011*	**0.284**	**0.416**	0.015*	0.019*
Residence location						
Urban	31.38 ± 40.79	30.88 ± 39.31	43.15 ± 35.33	40.93 ± 33.07	27.12 ± 7.11	57.57 ± 20.55
Rural	53.82 ± 72.78	51.53 ± 69.69	50.58 ± 57.74	45.98 ± 46.55	24.08 ± 6.70	50.52 ± 22.66
t	**− 2.267**	**− 2.176**	**− 0.933**	**− 0.763**	**2.774**	**2.096**
P	0.026*	0.032*	**0.354**	**0.447**	0.006**	0.038*
Education level, years						
< 6	58.30 ± 74.71	56.42 ± 71.46	46.36 ± 56.76	41.26 ± 44.88	23.93 ± 6.73	46.82 ± 20.38
6–9	33.66 ± 45.66	33.01 ± 45.25	42.08 ± 36.52	40.72 ± 34.21	24.60 ± 6.36	54.39 ± 21.21
9–12	21.28 ± 18.85	20.53 ± 16.66	45.78 ± 40.46	42.00 ± 35.14	28.26 ± 7.62	62.04 ± 20.72
> 12	34.23 ± 47.60	33.07 ± 43.90	51.08 ± 40.27	48.92 ± 37.74	29.58 ± 6.69	62.96 ± 20.55
H/F	**3.508**	**3.555**	**0.29**	**0.384**	**6.894**	**5.677**
P	0.017*	0.016*	**0.832**	**0.765**	0.000^**^	0.001*
Monthly household income						
< 1700	72.02 ± 79.75	70.20 ± 77.99	48.01 ± 50.98	47.20 ± 50.63	25.18 ± 5.95	53.03 ± 21.75
1701 ~ 5800	40.05 ± 55.48	38.72 ± 54.04	38.22 ± 37.10	35.68 ± 33.00	24.08 ± 6.97	49.68 ± 21.70
5801 ~ 15,000	30.02 ± 44.39	29.00 ± 39.54	58.23 ± 52.31	53.00 ± 40.55	29.20 ± 6.94	63.33 ± 18.36
> 15,000	26.31 ± 34.60	26.68 ± 34.53	30.88 ± 25.40	30.63 ± 25.93	24.08 ± 5.40	51.39 ± 24.83
H/F	**3.445**	**3.569**	**2.826**	**2.885**	**7.071**	**5.101**
P	0.018*	0.015*	0.040^*^	0.037^*^	0.000**	0.002**
Age						
18 ~ 40	50.43 ± 86.49	50.43 ± 86.49	57.82 ± 46.70	55.67 ± 45.91	27.27 ± 6.96	57.57 ± 19.88
41 ~ 65	38.09 ± 47.08	37.29 ± 45.73	42.24 ± 36.14	39.97 ± 33.35	25.91 ± 7.11	55.73 ± 21.98
≥ 65	40.87 ± 65.69	38.64 ± 60.84	51.45 ± 59.87	46.32 ± 46.90	25.9 ± 7.19	52.67 ± 21.19
H/F	**2.468**	**2.658**	**1.395**	**1.486**	**0.189**	**0.432**
P	**0.291**	**0.265**	**0.498**	**0.476**	**0.828**	**0.65**
Time of illness, month						
1 ~ 3	37.65 ± 48.93	37.37 ± 48.75	47.38 ± 40.77	45.35 ± 37.61	27.82 ± 7.43	57.53 ± 22.27
4 ~ 6	35.90 ± 50.85	34.91 ± 49.31	46.74 ± 40.41	43.98 ± 37.26	25.08 ± 6.88	56.05 ± 20.49
≥ 7	46.64 ± 69.02	38.52 ± 53.38	42.80 ± 54.98	37.78 ± 41.48	24.22 ± 6.27	50.00 ± 21.18
H/F	**0.439**	**0.387**	**0.164**	**0.597**	**4.551**	**1.901**
P	**0.803**	**0.679**	**0.849**	**0.552**	0.012*	**0.153**
Received chemoradiotherapy						
Yes	38.00 ± 50.29	36.72 ± 48.73	47.00 ± 44.28	44.07 ± 40.86	25.09 ± 6.66	58.51 ± 20.56
No	41.63 ± 61.68	40.60 ± 58.56	44.61 ± 45.90	41.33 ± 35.88	27.05 ± 7.47	50.83 ± 22.09
t	**− 0.427**	**− 0.476**	**0.349**	**0.465**	**− 1.828**	**2.366**
P	**0.67**	**0.635**	**0.727**	**0.642**	**0.069**	0.019^*^

*Statistically significant differences at p < 0.05

**Statistically significant differences at p < 0.01

### Associations between physical activity, self-efficacy, and quality of life

The results of the study showed a positive relationship between recreational PA and self-efficacy regardless of the season (P < 0.01). However, no statistically significant relationship was found between domestic PA and self-efficacy (Table 4).

**Table 4:** Associations between physical activity and self-efficacy of colorectal cancer survivors (January 2021–May 2021; N = 173)

Physical activit	Self-efficacy
Summer housework physical activity	0.124
Winter housework physical activity	0.123
Summer recreational physical activity	0.228**
Winter recreational physical activity	0.257**

**Statistically significant differences at p < 0.01

Furthermore, Self-efficacy scores were significantly and positively associated with function scores and global health status of QoL(P < 0.01). Conversely, self-efficacy scores were negatively associated with symptom scores (P < 0.05). Moreover, recreational PA scores were found to be positively associated with global health status (P < 0.05). (Table 5).

**Table 5:** Associations between self-efficacy, physical activity and quality of life (January 2021–May 2021; N = 173)

	Self-efficacy	Summer housework physical activity	Winter housework physical activity	Summer recreational physical activity	Winter recreational physical activity
Somatic function	0.398**	0.004	0.026	0.104	0.147
Role function	0.320**	0.107	0.114	0.125	0.145
Emotional function	0.410**	0.036	0.05	− 0.037	0.015
Cognitive function	0.243**	– 0.015	− 0.004	− 0.049	− 0.022
Social function	0.380**	0.043	0.065	− 0.016	0.046
Tiredness	− 0.362**	0.076	0.062	− 0.047	− 0.123
Nausea and vomiting	− 0.193*	− 0.013	− 0.015	0.029	0.025
Pain	− 0.246**	0.008	− 0.01	− 0.037	− 0.082
Shortness of breath	–0 .252**	− 0.037	− 0.044	− 0.053	− 0.082
Insomnia	− 0.205**	0.035	0.025	− 0.038	− 0.083
Anorexia	− 0.239**	− 0.07	− 0.078	− 0.048	− 0.057
Constipation	− 0.074	− 0.068	− 0.062	− 0.048	− 0.035
Diarrhea	− 0.239**	0.069	0.06	0.051	0.01
Economic hardship	− 0.286**	0.008	− 0.006	− 0.073	− 0.124
Global health status	0.458**	0.108	0.114	0.180*	0.230**

*Statistically significant differences at p < 0.05

**Statistically significant differences at p < 0.01

### Regression analysis of quality of life

In the hierarchical regression analysis, the QoL was considered the dependent variable. The independent variables included self-efficacy, PA, and general demographic data. The results from the analysis revealed that self-efficacy, recreational PA during winter, and a history of chemoradiotherapy accounted for 29.3% of the variance in QoL among survivors of colorectal cancer (CRC) (R^2^ = 0.293). (Table 6).

**Table 6:** Stratified regression analysis of quality of life (January 2021–May 2021; N = 173)

	Stratified analyses 1	Stratified analyses 2	Stratified analyses 3
Constant			
β			
*t*	3.373	3.386	4.867
*P*	0.001**	0.001**	0.000**
Self-efficacy			
β	0.458	0.416	0.543
*t*	6.739	5.969	6.649
*P*	0.000**	0.000**	0.000**
Summer recreational physical activity			
β		− 0.516	− 0.476
*t*		− 1.952	− 1.859
*P*		0.053	0.065
Winter recreational physical activity			
β		0.622	0.565
*t*		2.334	2.189
*P*		0.021*	0.030*
Chemoradiotherapy			
β			− 0.233
*t*			− 3.551
*P*			0.000**
n	173	173	173
* R*^2^	0.21	0.24	0.293

β = standardized coefficients

*Statistically significant differences at p < 0.05

**Statistically significant differences at p < 0.01

## Discussion

We explored the factors affecting PA, self-efficacy, and QoL in CRC survivors and conducted a regression analysis of the factors affecting the QoL to provide a reference for the development of interventions and guidelines for the development of interventions and strategies to improve their QoL. To the best of our knowledge, this is the first study to investigate the relationship between QoL, self-efficacy, and PA in CRC survivors. We found that both QoL and PA were influenced by self-efficacy. Our findings have important theoretical and practical implications. This can provide a theoretical basis and new ideas for further study of CRC and survivorship care planning for CRC survivors. The main findings are as follows:

First, CRC survivors had low levels of PA, self-efficacy, and QoL. Second, PA among CRC survivors was associated with sex, family residence, and self-efficacy. The global health status of CRC survivors differed significantly among sex, residence, level of education, whether they received chemoradiotherapy, and monthly income. Thirdly, self-efficacy, recreational PA, and chemoradiotherapy were the main factors affecting survivors’ health.

In our study, we observed a higher proportion of males compared to females among the participants. The age of the survivors was mainly concentrated in the 41–65 age group, and there were more urban dwellers. This was consistent with previous epidemiological evidence. Some studies have confirmed that processed food [24], alcohol [25], and overweight may increase the risk of CRC [26]. Urban and male survivors were more likely to have unhealthy lifestyles such as smoking, alcohol consumption, high-fat diet, and lack of PA.

We found that the majority of CRC survivors did not get enough physical activity and maintained a light to moderate level of PA, such as walking, swimming and housework, which is consistent with previous studies [13, 27, 28]. These results are unsurprising for several reasons. First, many CRC survivors may experience symptoms such as pain or rectal irritation that make it difficult to engage in more vigorous physical activity [29]. Second, postoperative drainage placement can lead to temporary physical limitations and poor health outcomes [30].

The PA of CRC survivors was influenced by many factors. For instance, female survivors tended to engage in more domestic physical activity than males, whether in summer or winter, which may be related to women's social roles. Women tend to do more housework. We also found that family income was associated with the amount of recreational physical activity that CRC survivors engaged in. Those with higher incomes tended to have more disposable time and financial resources to devote to leisure activities, which in turn led them to engage in more recreational physical activity. Additionally, it should be noted that the assessment of physical activity used in our study relied on self-reported measures, which may introduce certain biases, such as recall and interpretation biases.

Our study found a positive correlation between self-efficacy and recreational PA among CRC survivors. Those with higher levels of self-efficacy tended to engage in more recreational PA. Self-efficacy refers to an individual's belief in their ability to take action to achieve a certain goal or task, and our results suggest that having greater confidence in one's ability to exercise can lead to increased physical activity levels. However, the relationship between self-efficacy and PA is intricate. Participating in a specific level of physical activity can also enhance an individual's confidence and elevate their self-efficacy. In addition, educational level and family residence were also factors that influenced PA. This is consistent with the findings of Bao [13]. In addition, Kang et al. reported that the number of complications, fatigue, body image, depression, perceived benefits/barriers, and self-efficacy were closely correlated with PA [27]. A more comprehensive survey should be conducted in future research.

Our study showed that CRC survivors had lower levels of self-efficacy, which is consistent with previous studies [31]. Urban residents tended to have higher levels of self-efficacy, which may be related to better access to healthcare resources and supportive environments in cities. We also found that educational level was positively correlated with self-efficacy, likely because higher levels of education are associated with greater knowledge and understanding of the disease, as well as a greater ability to adopt healthy lifestyle habits [32]. However, we observed that self-efficacy tended to decrease over time as the illness became more chronic and symptoms worsened, leading to a loss of confidence and motivation. Increasing attention has been paid to the self-efficacy, however, there are few studies on CRC survival' self-efficacy. Future studies should continue to identify the factors associated with CRC survival’ self-efficacy.

The patient's general level of health was found to be low, consistent with Balhareth's findings [33]. Factors such as sex, residence, chemotherapy, monthly income, and education level could influence the QoL of CRC survivors. Survivors with higher monthly incomes and living in urban areas tend to receive better treatment and care, may resulting in a higher QoL. Additionally, survivors with higher level of literacy generally have more opportunities to understand the disease and adopt strategies for promoting health, leading to higher QoL scores. While chemotherapy is known to have toxic side effects, it can relieve symptoms and improve the QoL of survivors, especially in advanced stages of the disease. However, it is worth noting that chemotherapy is often correlated with the severity of the disease, and the severity of the disease, in turn, can have an impact on the QoL experienced by patients. The relationship between these factors is highly intricate and multifaceted.

Recreational PA was found to positively affect survivors' general health. Engaging in leisure-time activities, such as playing basketball, is associated with better mood and higher intensity, while domestic PA may have the opposite effect. Furthermore, improved mood is linked to higher scores on the mental health dimension, indicating a higher overall QoL with more recreational PA. This is consistent with previous researches [34–37]. However, it is important to consider that certain recreational physical activities often demand a certain level of physical fitness, and an individual's physical fitness is closely related to the severity of their disease. It has also been reported that physical activity has an indirect effect on QoL over time through self-efficacy and health status [38, 39]. Whether self-efficacy in CRC survivors is mediated in this way requires further research. Hidde's study reported no significant association between isotemporal substitution effects for reallocating time between different types of PA and QoL, but the specific types of activity were not specified [40]. However, the type of activity is not specified. Therefore, determining the optimal type and intensity of PA remains a challenge.

Self-efficacy has been found to have a positive impact on QoL. On the one hand, self-efficacy represents the patient's confidence in overcoming the disease, which can directly affect the patient's psychological state and have a certain impact on their overall health status [41]. On the other hand, survivors with higher self-efficacy are more likely to engage in self-management and take appropriate actions to promote health and improve QoL [42]. Our results were consistent with Lee et al. They found that providing enough additional information to improve self-efficacy could promote healthy lifestyles, thereby improving QoL [43]. However, there are few studies on the impact of self-efficacy on QoL in CRC survivors. The mechanisms and effects of self-efficacy, and in particular the mediating role of self-efficacy, should be explored in greater depth in future research.

At the same time, Measures should be considered from the aspects of self-efficacy and PA to improve the QoL of CRC survivors. Individualized PA programs should be developed to enhance CRC survivors’ physical and mental recovery, thereby improving their overall QoL.

### Clinical implications

CRC survivors have low QoL, which inflict a heavy economic and psychological burden on families and society [44, 45]. The increase in PA can improve QoL of CRC survivors [9, 46–48]. We found a strong correlation between the type of physical activity, self-efficacy, and QoL, especially recreational PA. Our study may therefore inform clinical practice in cancer survivors’ care. Personalized cancer care plans are important. Future development of survival plans for CRC survivors should take into account the type of PA, such as Baduanjin [37], yoga [49, 50], etc., and develop an intervention plan that incorporates the patient's preferred activities.

Our study also found the importance of self-efficacy, which is low in CRC survivors, but has a positive impact on both PA and QoL [51, 52]. Our study could provide theoretical guidance for CRC survivorship interventions. It is necessary to consider survivors' self-efficacy when developing CRC survivorship intervention plans. Survivors should receive adequate social support from their surroundings, such as encouragement and support from relatives and close friends, as well as informative support from medical staff, to increase their confidence in overcoming the disease, thereby increasing their self-efficacy, improving their QoL and enhancing the effectiveness and quality of care.

## Limitation

This study is also subject to limitations. Firstly, although self-report measures provide an easy, accessible, and practical method, but they are subject to recall bias. Secondly, the directionality of the relationship and causal relationship between change in PA, self-efficacy, and QoL cannot be determined due to the cross-sectional study design. In addition, the samples were not representative, as they were recruited from the three local hospitals, and therefore selection bias could not be ruled out.

## Conclusion

In conclusion, the findings of this study highlight that CRC survivors had low levels of PA, self-efficacy and QoL. Self-efficacy influenced PA and QoL, but the causal relationship was unclear. More high-quality research is needed to explore the role of self-efficacy and whether it is a mediator of PA and QoL. Future cancer survivors care can make full use of self-efficacy, unleashing survivors' potential and initiative, and increasing survivors' confidence in their treatment. When devising intervention programs for CRC survivors, it is important to develop personalized plans that consider individual factors, such as gender and educational level. This personalized approach will not only enhance the effectiveness of the interventions but also foster better cooperation and engagement from the individuals themselves.

Moreover, future studies should continue to explore additional factors that influence the QoL of cancer survivors. By incorporating a broader range of variables into the analysis, we can gain a more comprehensive understanding of the factors affecting the QoL of colorectal cancer survivors. These endeavors aim to improve the overall well-being and QoL of individuals who have successfully overcome CRC.

## Data Availability

The datasets generated during and analysed during the current study are available from the corresponding author on reasonable request.
